# *Streptococcus mutans* and Caries: A Systematic Review and Meta-Analysis

**DOI:** 10.1177/00220345241303880

**Published:** 2025-02-02

**Authors:** D. Mazurel, B.W. Brandt, M. Boomsma, W. Crielaard, M. Lagerweij, R.A.M. Exterkate, D.M. Deng

**Affiliations:** 1 Department of Preventive Dentistry, Academic Center for Dentistry Amsterdam (ACTA), University of Amsterdam and VU University Amsterdam, Amsterdam, Noord-Holland, The Netherlands; 2 Department of Cariology, Academic Center for Dentistry Amsterdam (ACTA), University of Amsterdam and VU University Amsterdam, Amsterdam, Noord-Holland, The Netherlands

**Keywords:** microbiota, 16s ribosomal rna, high-throughput nucleotide sequencing, real-time polymerase chain reaction, dental plaque, saliva

## Abstract

It has been questioned whether *Streptococcus mutans* can still be considered the major etiological agent for caries. The main argument is that most evidence has been based on single-species identification. The composition of the oral microbiome was not analyzed. This systemic review aims to assess the prevalence and abundance of *S. mutans* in caries-active (CA) and caries-free (CF) subjects based on clinical studies in which the microbiome was investigated. Three databases (PubMed, Cochrane, Embase) were searched until May 22, 2023, for eligible publications that included CA and CF subjects and reported the detection of both *S. mutans* and the oral microbial community, using DNA-based methods. The clinical and microbial outcomes were summarized and further analyzed using a random-effects model. Of 22 eligible studies, 3 were excluded due to the high risk of bias. In the remaining 19 studies, 16 reported the prevalence of *S. mutans*, 11 reported its relative abundance, and 8 reported both parameters. The prevalence of *S. mutans* in CA was either similar to (*n* = 4) or higher than (*n* = 12) the CF group. The reported relative abundance in CA was higher than CF in all 11 studies, although the values varied from 0.001% to 5%. Meta-analysis confirmed the significance of these findings. The summary of microbial community data did not reveal other caries-associated bacterial genera/species than *S. mutans*. In conclusion, the collected evidence based on microbiome studies suggests a strong association between the prevalence and abundance of *S. mutans* and caries experience. While the cariogenic role of *S. mutans* in the oral ecosystem should be recognized, its actual function warrants further exploration.

## Introduction

Caries is characterized by the net mineral loss of dental hard tissues, resulting from an imbalance between demineralization and remineralization of the tooth’s surface. The breakdown of mineral equilibrium is caused by the accumulation of organic acid produced by oral bacterial during carbohydrate fermentation (Takahashi and Nyvad 2008; Marsh and Zaura 2017). Identified by J. Kilian Clarke in 1924, *Streptococcus mutans* has been extensively studied and considered as the major etiological agent driving the mineral equilibrium breakdown (Clarke 1924; Loesche 1986; Lemos et al. 2019). It is both acidogenic, with a strong organic acid production capacity, and aciduric, which enables it to persist under prolonged periods of low pH (Lemos et al. 2019; Bedoya-Correa et al. 2021). Its ability to produce extracellular matrix strengthens its persistence in biofilms (Lemos et al. 2019; Bedoya-Correa et al. 2021). Hence, it is thought that *S. mutans* possessed competitive advantages within dental biofilms and had a high cariogenic potential (Lemos et al. 2019). Considerable research efforts have been dedicated to unraveling the genetic mechanisms dictating its acidogenicity, stress tolerance, biofilm formation, and signaling pathways. Consequently, *S. mutans* has been used as a microbial caries marker for caries risk assessment and prognosis in vivo (Cagetti et al. 2018). The development of a novel caries-preventive agent has frequently centered on targeting the eradication or inhibition of *S. mutans* (Lemos et al. 2019).

Despite the vast achievements in our knowledge on *S. mutans*, doubts have emerged regarding its cariogenic role and its suitability as a model microorganism for evaluating caries-preventive strategy (Banas and Drake 2018; Philip et al. 2018). These uncertainties have arisen due to a deeper understanding of the plaque ecology concept, alongside the inconclusive findings observed in clinical research (Dinis et al. 2022). While some clinical studies have illustrated a causal relationship between the levels of *S. mutans* in plaque or saliva and caries experience (Saraithong et al. 2015; Damle et al. 2016; Edelstein et al. 2016), others have failed to establish this correlation (Carlsson et al. 1985; Baca et al. 2011). Furthermore, studies have reported undetectable levels of *S. mutans* in subjects with rampant caries (Aas et al. 2008; Belda-Ferre et al. 2012; Dinis et al. 2022), while high levels of *S. mutans* have been observed in healthy subjects (Kahharova et al. 2020). In 1994, an ecological plaque hypothesis was proposed, emphasizing that dental caries is caused by a shift in the balance of a microbial community rather than the presence of a specific bacterial species (Marsh 1994). The advent of state-of-art next-generation sequencing technology has enabled researchers to validate this ecological plaque hypothesis and decipher the oral microbiome (Marsh and Zaura 2017). Subsequently, multiple clinical studies have investigated not only the prevalence of *S. mutans* but also the composition of oral microbiome in caries-free (CF) and caries-active (CA) populations. These investigations facilitate a reexamination of the association between *S. mutans* levels and caries status, based on clinical data that incorporate the oral microbial community. The aim of this study is to systematically assess the prevalence and abundance of *S. mutans* in CF and CA individuals in clinical studies in which the composition of oral microbiome was also investigated.

## Methods

This systematic review was registered (CRD42022293345) at the International Prospective Register of Systematic Reviews (PROSPERO) and performed according to the guidelines of Preferred Reporting Items for Systematic Reviews and Meta-analyses (PRISMA) (Page et al. 2021).

### Search Strategy and Inclusion and Exclusion Criteria

The search strategy (Appendix Table 1) and inclusion criteria were formulated according to PECOS format (Participants, Exposure, Comparisons, Outcomes, and Study design): studies were included when systematic healthy individuals (P) with caries (E) were compared with CF individuals (C) on frequency or abundance of *S. mutans* (O) in oral samples. The studies must have also analyzed/reported the composition of the oral microbial community (with at least 40 bacterial species) using a DNA/RNA-based method (O). The studies could be case-control, clinical intervention, or longitudinal studies (S).

Exclusion criteria were (1) case report/case series; (2) studies including participants who had a systemic disease, or were pregnant, or whose systemic health was not reported; (3) studies including participants who used medication (e.g., antibiotics) within the past month or whose medication status was not mentioned; (4) intervention studies with no baseline data; and (5) studies including oral samples taken from any foreign materials such as bonding agent, orthodontic appliances/brackets, or dentures.

Relevant literature was identified by searching the PubMed, Cochrane, and Embase databases until May 22, 2023. Screening was performed by 2 independent researchers (D.M. and M.B.) using Rayyan QCRI (Ouzzani et al. 2016). Articles were first screened for eligibility by their titles and abstracts and then by full text. Disagreements were resolved by consensus-based discussion between D.M. and M.B. or by a third researcher (D.D.).

### Quality Assessment

The quality of the included articles was assessed by 2 independent researchers using the 10-item Joanna Briggs Institute (JBI) Critical Appraisal tools for systematic Reviews (Moola et al. 2020). Methodological features of the studies were evaluated and scored positively (“Yes” or “Not applicable”) or negatively (“No” or “Unclear”) per item. Each positively evaluated item was given 1 point to the total JBI score of the individual study. Studies scoring ≤6 were considered as low quality and with high risk of bias. The studies with high risk of bias were excluded from the downstream data synthesis and meta-analysis.

### Data Synthesis and Meta-analysis

Data synthesis of the selected studies included extraction of general characteristics, such as number and age of participants, sample type, and extraction of primary outcome variables. These variables include the prevalence and/or relative abundance of *S. mutans* and reported composition of the microbial community. The prevalence refers to the percentage of participants carrying *S. mutans*. The relative abundance refers to the sequencing reads of *S. mutans* relative to the total reads of detected bacterial species. The data on prevalence and relative abundance were either obtained directly from the publications or recalculated from available data.

Meta-analysis was performed to compare the prevalence and relative abundance of *S. mutans* between CA and CF subjects, using Review Manager (RevMan) version 5.4.1 (Review Manager [RevMan] 2020). Odds ratios and standard mean differences were calculated using the Mantel-Haenszel test for random effects or the inverse variance test for random effects, for prevalence and relative abundance, respectively, with a 95% confidence interval. In addition, heterogeneity among studies was assessed using the χ^2^ test (with *P* < 0.10 indicating a significant heterogeneity) and the *I*^2^ (with 30% to 100% indicating moderate up to considerable heterogeneity).

## Results

**Figure 1.**
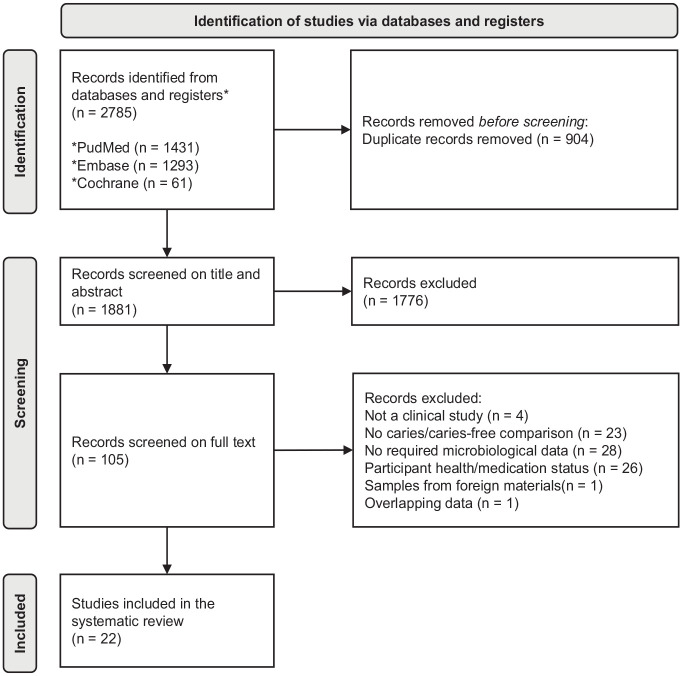
PRISMA flow diagram of the study selection.

The search query initially yielded 1,881 unique records. After the title and abstract screening, 105 studies were selected. Full-text screening of these studies resulted in 22 studies for quality assessment (Fig. 1). The detailed explanations of full-text article exclusion are listed in Appendix Table 2. Beside the exclusion criteria mentioned in the Methods, 1 article (Eriksson et al. 2018) was excluded to prevent any overlap of data. This study was conducted by the same research group and used the same but smaller cohort as the other included study (Eriksson et al. 2017).

### Quality Assessment

The individual score per JBI item per article is shown in Appendix Table 3. Of 22 studies, 12 scored 10, with a low risk of bias; 7 scored between 7 and 9, with a medium risk of bias; and 3 scored 6, with a high risk of bias (Kouidhi et al. 2014; Hurley et al. 2019; Zheng et al. 2021). The main negative assessments are (1) the CA and CF groups were not matched and (2) strategies to deal with confounding factors, such as randomization or statistical testing, were not stated. The studies with a high risk of bias (*n* = 3) were excluded from subsequent data synthesis and meta-analysis.

### Study Characteristics

Appendix Table 4 shows the characteristics of 19 included studies. These studies included participants from 7 different countries: 8 in China, 5 in the United States, 2 in Sweden, 1 in Denmark, 1 in Australia, 1 in Kuwait, and 1 in Thailand. The age of the subjects ranged from 40 mo to 50 y, and the number of subjects per study was 20 to 154. The sample types were either saliva and/or supragingival plaque. Sampling methods varied among studies: saliva samples were collected as stimulated (by paraffin wax chewing) or nonstimulated (by drooling, spitting, pipetting, or pumping), and plaque samples were pooled from multiple surfaces including or excluding carious lesions. Different methods have been used to determine the microbial compositions: 16S rRNA gene amplicon sequencing (*n* = 12), whole-genome shotgun sequencing (*n* = 4), Sanger sequencing (*n* = 1), and the Human Oral Microbe Identification Microarray (HOMIM) (*n* = 2). Among the studies using 16S rRNA gene amplicon sequencing methods, various regions of the 16S rRNA regions were targeted: V1-V3 or V1-V2 (*n* = 3), V4 or V3-V4 (*n* = 7), and V1-V9 (*n* = 2).

### Clinical Diagnosis of Caries

The clinical diagnosis of caries and the severity of caries were assessed across 19 studies, grouped by detection level (Table 1). Caries detection was reported either per tooth, per tooth surface, or both, with dentition type including primary, permanent, or mixed. Various diagnostic criteria were used, such as the American Academy of Pediatric Dentistry criteria for severe early childhood caries (S-ECC; *n* = 7), the International Caries Detection and Assessment System (ICDAS II; *n* = 2), and self-defined criteria (*n* = 10) (International Caries Detection and Assessment System [ICDAS] Coordinating Committee 2009; American Academy of Pediatric Dentistry 2022). The self-defined criteria for CA subjects varied from DFT ≥ 1 to dmft/DMFT ≥ 7. In addition, 3 studies considered a subject to be CA only when an active carious lesion was present at the time of inclusion (Belstrøm et al. 2017; Dashper et al. 2019; Baker et al. 2021). In contrast, the definition of CF subjects was consistent among 19 studies. Fifteen required an absence of any decayed, missing, of filled surfaces or teeth, and the remaining 4 required only no active carious lesions without specifically excluding missing and/or filled teeth (Tanner et al. 2011; Belstrøm et al. 2017; Eriksson et al. 2018; Dashper et al. 2019). The caries severity varied greatly among studies. For example, at the tooth level, the dmft/DMFT varied from 4.0 to 11.4.

**Table 1.** Severity of Caries and the Applied Clinical Diagnostic Criteria among Selected Studies.

Caries Severity (Mean ± SD)^a^			Clinical Diagnostic Criteria^b^	Dentition Type	Study
Detection Level	dmft/DMFT^c^	dmfs/DMFS^c^			
Tooth and surface	11.4 ± 2.4	24.7 ± 10.5	S-ECC	Primary	Tang et al. 2022
	10.3 ± 5.1	25.2 ± 13.9	S-ECC	Primary	Wu et al. 2021
	4.4 ± 2.3	7.9 ± 2.2	dmft ≥ 2	Primary	Zheng et al. 2018
	4.1 ± 0.1	4.9 ± 1.6	dmft/DMFT ≥ 3	Mixed	Yang et al. 2021
Tooth	11.4	—	DS > 3	Permanent	Belstrøm et al. 2017
	10.6 ± 3.0	—	dmft/DMFT ≥ 5 and dt/DT ≥ 2	Mixed	Qudeimat et al. 2021
	8.8 ± 1.7 - 9.7 ± 2.2	—	dmft/DMFT ≥ 6	Mixed	Li et al. 2021
	8.8 ± 3.8	—	≥2 dentin caries	Mixed	Baker et al. 2021
	8.8	—	S-ECC	Primary	Garcia et al. 2021
	6.8 ± 0.9	—	DMFT ≥ 6	Permanent	Zheng et al. 2017
	4.0 ± 3.8	—	dmft ≥ 2	Primary	Jiang et al. 2016
	—	—	DMFT ≥ 7 (ICDAS 3–6)	Primary	Wang et al. 2022
Surface	—	33% (%ds)^d^	S-ECC	Primary	Kanasi et al. 2010
	—	32.4% ± 2.7 (%ds)^d^	S-ECC	Primary	Tanner et al. 2011
	—	17.25 (DFS)	DFT ≥ 3	Permanent	Havsed et al. 2021
	—	11.1 ± 5.6	S-ECC	Primary	Ma et al. 2015
	—	5.9 (DS/DFS)	DFT ≥ 1	Permanent	Eriksson et al. 2017
	—	4.4 ± 5.6	S-ECC	Primary	Wu et al. 2022
	—	—	ds ≥ 1 (ICDAS II ≥ 2)	Primary	Dashper et al. 2019

a Studies are grouped by the level at which the caries lesion was detected (tooth and surface level, tooth level, or surface level) and ordered from high to low caries severity within each detection level.

b The clinical diagnostic criteria for the caries-active group. S-ECC, severe early childhood caries diagnosed by the American Academy of Pediatric Dentistry criteria (Drury et al. 1999; American Academy of Pediatric Dentistry 2022). ICDAS II, the Modified International Caries Detection and Assessment System (ICDAS II) (International Caries Detection and Assessment System (ICDAS) Coordinating Committee 2009).

c dmft/s or DMFT/S: decayed (d), missing (m), filled (f), teeth (t) or surfaces (s). Lowercase letters indicate primary dentition; uppercase letters indicate permanent dentition. —, not reported.

d For Kanasi et al. (2010) and Tanner et al. (2011), severity was reported as a percentage of decayed surfaces relative to the total erupted surfaces.

### Prevalence and Relative Abundance of *S. mutans* in the CA and CF Groups

**Figure 2.**
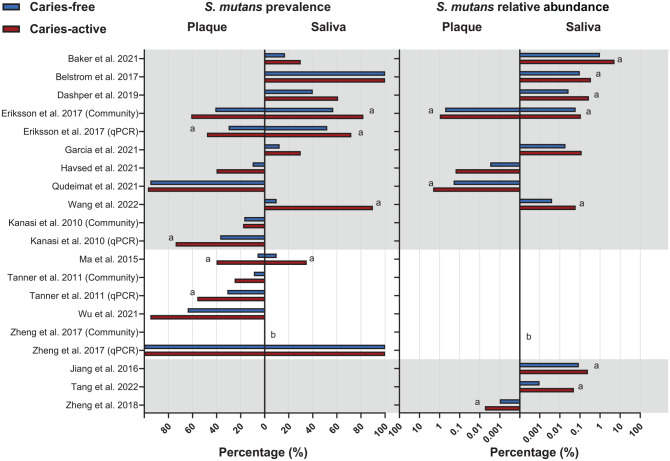
Prevalence (%) and relative abundance (%) of *S. mutans* in the saliva and plaque of caries-active and caries-free individuals. Studies are listed in alphabetical order and grouped by reported outcomes (both prevalence and relative abundance, prevalence only, relative abundance only). Studies (*n* = 3) that did not report mean values in the publication are not included in this graph. Prevalence is shown on a linear scale, whereas the relative abundance is shown on a log10 scale. (**a**) A significant difference was reported in the study. (**b**) *S. mutans* was not detected.

The prevalence (*n* = 13) and relative abundance (*n* = 11) of *S. mutans* in plaque or saliva samples were assessed in both CA and CF groups, with 8 studies reporting both parameters (Fig. 2). All data were either directly retrieved from the publications or calculated based on the reported or acquired data. Of the 19 studies reviewed, data from 3 studies (Li et al. 2021; Yang et al. 2021; Wu et al. 2022) could not be retrieved, although multiple requests were sent to the authors. Consequently, the data related to the prevalence and abundance of *S. mutans* in these 3 studies were excluded in Figure 2 and downstream meta-analysis.

The prevalence of *S. mutans* in CA was either similar to (*n* = 4) or higher than (*n* = 9) that in CF irrespective of sample type, although it varied from 10% to 100% among studies. The 3 studies without data also claimed that the prevalence of *S. mutans* in CA was significantly higher than in CF. Similarly, the reported relative abundance of *S. mutans* in CA was higher than that in CF (*n* = 11), although it varied from 0.001% to 5% among studies. Nine out of 11 studies stated that the difference in relative abundance was statistically significant. Interestingly, 2 studies that did not find a difference in *S. mutans* prevalence reported that *S. mutans* was significantly more abundant in CA than in CF (Belstrøm et al. 2017; Qudeimat et al. 2021).

Among 19 studies, 4 performed additional species-specific quantitative polymerase chain reaction (qPCR) besides sequencing or microarray. Table 2 illustrates the comparisons of *S. mutans* detection based on the microbial community detection methods or species-specific qPCR. In the study by Eriksson et al. (2017), the qPCR results were in line with those by Illumina sequencing, but in the other study by Zheng et al. (2017), the qPCR results were very different from those obtained by Illumina sequencing. With qPCR, *S. mutans* was detected in all subjects, whereas no *S. mutans* was detected with sequencing. The 16S rRNA gene target region for Illumina sequencing was different in these 2 studies: one targeted the V3-V4 region and the other the V1-V2 region. In Kanasi et al. (2010) and Tanner et al. (2011), the prevalence of *S. mutans* by qPCR was twice as high as that by the community detection methods, clonal analysis, and HOMIM microarray (Kanasi et al. 2010; Tanner et al. 2011).

**Table 2.** Detection of *S. mutans* in the CA and CF Groups, as Determined by Both Microbial Community Detection Method and Species-Specific qPCR Detection.

Study	Sample Type	*S. mutans* Detected by Microbial Community Detection Method		*S. mutans* Detected by qPCR
		Platform (RNA/DNA Target Region)	Prevalence (CA/CF)	Prevalence (CA/CF)
Eriksson et al. 2017	Saliva	Illumina MiSeq (16S rRNA region V3–V4)	82%/57%^a^	72%/52%^a^
	Plaque		61%/41%	48%/30%^a^
Kanasi et al. 2010	Plaque	Clonal analysis and Sanger sequencing (full 16S rRNA)	18%/17%	74%/37%^a^
Tanner et al. 2011	Plaque	HOMIM microarray (16S rRNA probes)	25%/9%	56%/31%^a^
Zheng et al. 2017	Saliva	Illumina MiSeq (16S rRNA region V1–V2)	0%/0%	100%/100%

CA, caries active; CF, caries free; qPCR, quantitative polymerase chain reaction.

a Significant difference between the CA and CF groups was reported in the study.

### Meta-Analysis of *S. mutans* Prevalence and Relative Abundance

**Figure 3.**
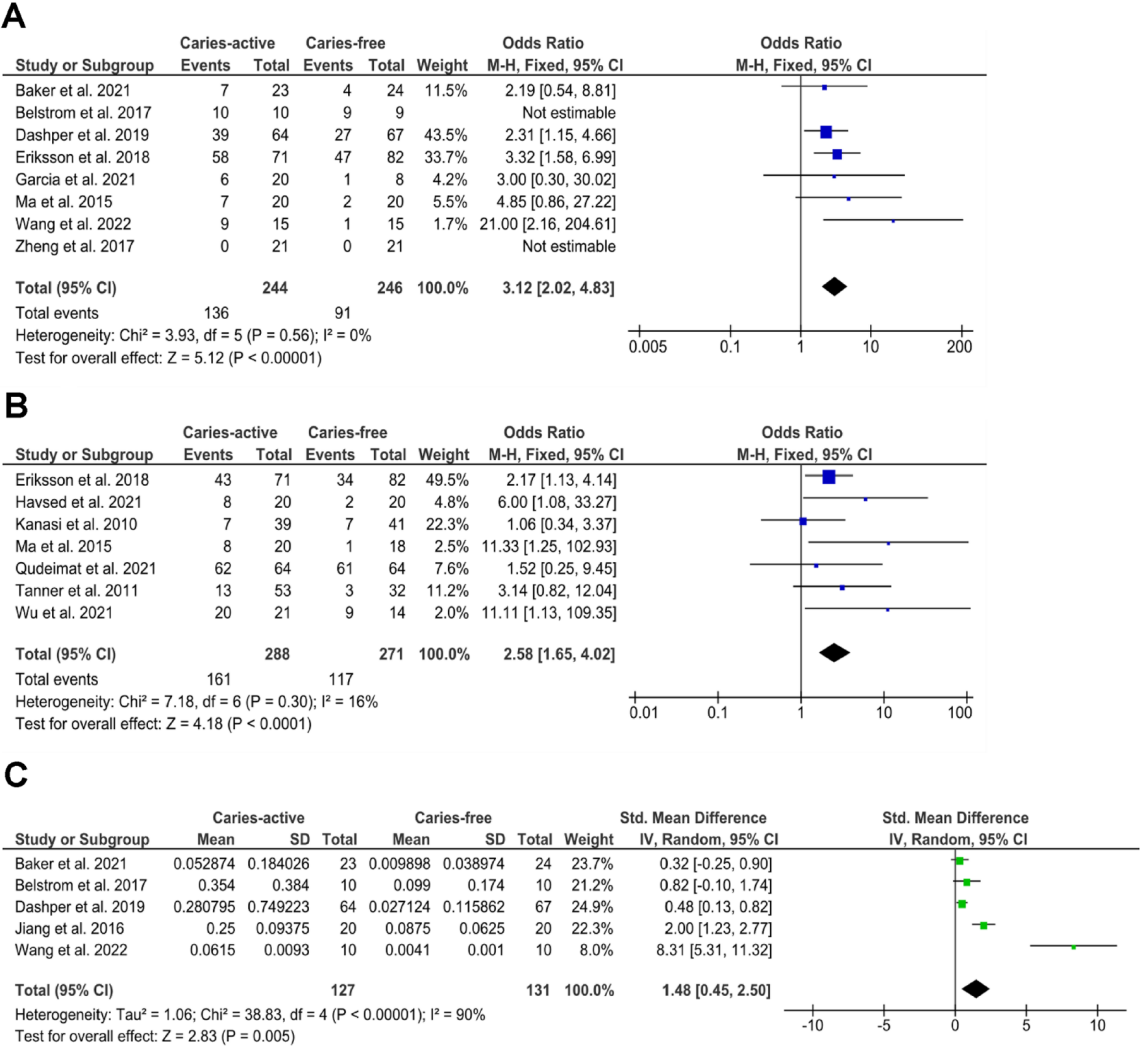
Meta-analysis of the *S. mutans* (**A**) prevalence in saliva, (**B**) prevalence in plaque, and (**C**) relative abundance (in %) in saliva, in caries-active versus caries-free individuals.

Meta-analysis per sample type (saliva or plaque) was further performed to examine the differences in *S. mutans* prevalence and relative abundance between the CA and CF groups (Fig. 3). For prevalence, Figure 3A and B show that the odds of detecting *S. mutans* is significantly higher in CA than CF in saliva (*P* < 0.0001) and in plaque (*P* < 0.0001). The heterogenicity of the data was very low, with an *I*^2^ of 0% (saliva) and 16% (plaque), indicating the high consistency in the CA-CF comparison among studies. For relative abundance, 5 of 9 studies with saliva samples reported or provided standard deviation values required for meta-analysis; hence, they were included in the analysis. Figure 3C shows that the relative abundance of *S. mutans* was also significantly higher in the CA group than in the CF group (*P* = 0.005). However, the heterogenicity of the data was rather high, with an *I*^2^ of 90%.

### Composition of the Oral Microbiome in the CA and CF Groups

Since we were unable to retrieve the sequencing data or operational taxonomic unit table of the oral microbiome from all included studies, the summaries below are based on the reports provided in the articles.

First, we extracted the 5 most abundant genera from 15 of 19 studies (Appendix Fig. 1). The genera shared by saliva and plaque samples were *Streptococcus*, *Veillonella*, *Neisseria*, *Prevotella*, *Leptotrichia*, *Capnocytophaga*, and *Actinomyces*. The site-specific genera were *Corynebacterium* and *Fusobacterium* for plaque and *Granulicatella*, *Haemophilus*, *Porphyromonas*, and *Rothia* for saliva.

Next, we summarized the bacterial species reported at significantly higher abundances in CA individuals (Table 3). The species mentioned in at least 2 different studies per sample type are listed. Since the individual bacterial species varied greatly among studies, we summed the species at the genus level. The genera of *Streptococcus*, *Prevotella*, and *Lactobacillus* were most often reported as significantly associated with CA, irrespective of the sample type. Within the genus *Streptococcus*, *S. mutans* was reported to have higher relative abundance in the CA than in the CF group in 10 of the 11 studies (saliva) and all 4 studies (plaque). No other individual species of *Streptococcus* or other genera were identified by more than 2 studies.

**Table 3.** Bacterial Species with Higher Relative Abundance in Caries-Active Individuals^a^.

	Number of Studies^c^	
Genus (Specified Species)^b^	Plaque	Saliva
*Actinomyces* (*gerencseriae*, *graevenitzii*, IP073, *massiliensis*, oral taxon 414, oral taxon 448)	—	3
*Dialister* (*invisus, micraerophilus, pneumosintes*)	2	—
***Lactobacillus*** (*crispatus, fermentum, gasseri, rhamnosus, oris*, unclassified, *vaginalis*)	**2**	**4**
*Leptotrichia* (*buccalis*, HOT498, *shahii*, IK040)	2	—
*Neisseria* (*flavescens, sicca*)	2	—
***Prevotella*** (*denticola, histicola, melaninogenica, oris, salivae*, unclassified, HOT301, *maculosa*)	**4**	**6**
*Propionibacterium* (*acidifaciens*, FMA5, unclassified)	—	4
*Rothia* (*dentocariosa, mucilaginosa*)	—	2
***Streptococcus*** (*anginosus, downei, gordonii, mutans, parasanguinis, sanguinis, sobrinus*, C150)	**4**	**11**
*Treponema* (HOT270, *vincentii*)	2	—
*Veillonella* (*atypica*, *dispar*, oral taxon 780)	3	—

a The listed bacterial species exhibited higher relative abundance in caries-active individuals compared with caries-free individuals, as reported in at least 2 different studies per sample type.

b The bacterial species are summarized at the genus level. The bacterial species mentioned in the included studies are listed in brackets. The text in bold highlights the 3 most reported genera.

c Number of studies reporting the indicated genus. — indicates that the genus was reported in ≤1 study.

We also extracted the reported α-diversity and β-diversity and examined if they were able to differentiate the CA and CF groups. Appendix Table 5 summarizes the findings retrieved from 14 of 19 studies. In terms of α-diversity, ~87% of comparisons did not find any significant differences between the CA and CF groups. The β-diversity was less reported. Among the studies reporting β-diversity, 38% to 60% found no difference between the CA and CF groups.

## Discussion

Dental caries is a multifactorial disease, caused by a dysbiotic microbial community rather than a single or few cariogenic bacterial species. The notion of *S. mutans* as the primary etiologic agent has been questioned (Banas and Drake 2018; Philip et al. 2018). This systematic review focuses on clinical studies that not only evaluated the association between *S. mutans* level and caries experience but also analyzed the composition of the microbial community in the same sample. We identified 19 studies with low or medium risk of bias. These studies revealed that the prevalence and/or relative abundance of *S. mutans* in CA groups were higher than those in CF groups. Subsequent meta-analysis further confirmed this association with strong statistical significance. Notably, the systematic summary of the community data did not identify any other bacterial species consistently associated with caries experience. Importantly, all included studies analyzed pooled plaque or saliva samples instead of site-specific samples. Despite this, the strong association between *S. mutans* and caries experience remained evident, underscoring the robustness of this relationship even without site-specific sampling.

In a recent review, Banas et al. (2018) pointed out the difficulties in determining a “cariogenic microbiome” based on the existing literature, due to the inconsistency among clinical studies. In the current study, we indeed recognized enormous variations among the clinical studies. Besides the variations already mentioned by Banas et al.—sampling choices (saliva or dental plaque; plaque collection from pooled or caries sites) and microbiome determination (various sequencing methods)—the most striking variation is the differences in clinical caries diagnosis. Among 19 studies, 10 used self-defined criteria for the CA group, which varied from DFT ≥ 1 to dmft/DMFT ≥ 7. This lack of standardization in caries diagnosis and clinical data/sample collection has been reported by other studies as well, which aimed to identify microorganisms associated with cariogenic dysbiotic biofilms (Dame-Teixeira et al. 2021) or investigated the prevalence of caries in children of different ages (Amarante et al. 1998). Despite the enormous variations in the clinical diagnostics and methodologies in all selected studies, our data synthesis revealed a consistent finding: high levels of *S. mutans* were associated with the CA population. This strengthens the previous notion on the cariogenic role of *S. mutans* in the oral microbial community (Belda-Ferre et al. 2012; Damle et al. 2016; Edelstein et al. 2016).

Another notable variation among the studies is the sequencing procedures used. Of the 19 studies reviewed, 12 used 16S rRNA gene amplicon sequencing for microbial profiling. Variations in this method were evident at several stages, including DNA extraction, selection of specific hypervariable regions for primer design, and databases used for taxonomy assignment. Previous research indicated that the choice of the DNA extraction method and the hypervariable regions of the 16S rRNA gene significantly influence the final profiling of oral microbiota biodiversity (Teng et al. 2018). In addition, another study suggested that the poor performance of a certain hypervariable region could be attributed to the reference database used (Abellan-Schneyder et al. 2021). In this systematic review, 4 studies incorporated additional qPCR to validate the presence of *S. mutans*, allowing for a comparative evaluation of the sequencing methods used across different studies. However, only 2 of the 4 studies performed the 16S rRNA sequencing method: one study targeting the V3–V4 region demonstrated a good correlation with qPCR for *S. mutans* identification, whereas the other study targeting the V1–V2 region found no *S. mutans* in any samples, despite qPCR identifying it in 100% of cases. Due to the small number of studies and the complexity of sequencing procedures, we cannot make definitive recommendations regarding primer regions for identifying *S. mutans*. Nevertheless, it has been advised to include a mock community in the sequencing process, as well as additional validation steps such as qPCR, and the use of 2 data-processing pipelines to enhance the credibility of microbial profiling (Teng et al. 2018; Bergsten et al. 2020).

Despite the high prevalence of *S. mutans* in CA subjects, it is worth noting that the reported relative abundance of *S. mutans* in a microbial community was rather low, being 0.001% to 5% of the total taxa. Until now, there has been no consensus on the definition of low-abundance species in a microbiota. Some studies considered 5% to 20% as low abundance, whereas the others consider 1% to 5% or <1% as low abundance (Cena et al. 2021). In microbiome studies, the low-abundant taxa are often filtered out during data processing or analyses. However, the mediator roles of low-abundant bacteria in microbial communities have been increasingly recognized (Han and Vaishnava 2023). For example, these bacterial species were shown to maintain the microbial community structure (Arumugam et al. 2011) or restore the structure after antibiotic treatments (Hildebrand et al. 2019). In fact, the modulating role of low-abundance *S. mutans* has been reported in previous studies. Xiao et al. (2012) showed that the extracellular polysaccharides produced by *S. mutans* regulated the mixed-species biofilm community by shaping biofilm architecture, enhancing local acidity, and promoting conditions favorable for caries development. Dinis et al. (2022) investigated the influence of *S. mutans* abundances on salivary microbiome composition and caries progression. They discovered that disease-associated species were significantly increased when the relative abundance of *S. mutans* was 0.393%. This evidence illustrated the potential driver role of *S. mutans* in shifting a microbial community toward dysbiosis. Unfortunately, the clinical evidence gathered in this study only suggested a strong association between *S. mutans* and caries experience, as direct correlation between the plaque or saliva samples and caries formation could not be established in the cross-sectional studies. Further research is needed to illustrate the role of *S. mutans* in microbial communities and caries formation.

While our primary focus was not to identify novel parameters or bacterial species that are associated with caries experience, we summarized the reported compositional data of the microbiome. Differential bacterial genera were reported in saliva and dental plaque samples, consistent with the well-established knowledge that the structure of the oral microbiome is site specific (Mark Welch et al. 2019). However, we were not able to identify any new cariogenic biomarkers based on the community data reported due to large variations among reports. Regarding the α-diversity of the microbial community, only 2 studies reported differences between the CA and CF groups. On the other hand, half of the studies reported differences in β-diversity, and the remaining half did not. Regarding CA-associated abundant bacterial species, a wide array of taxa was identified across the included 19 studies. At the bacterial genus level, *Streptococcus* emerged as the primary CA-associated taxon, with *S. mutans* showing the strongest association. This finding aligns with a scoping review by Bhaumik et al. (2021), in which 14 of 24 studies identified the association of *S. mutans* with caries. Despite these consistent observations, identifying definitive caries-associated or health-associated bacterial species remains challenging.

In this study, we aim to assess the levels of *S. mutans* in CA and CF subjects when the composition of microbial community was analyzed in the same study. Therefore, *S. mutans* was included as a keyword in the search query. A limitation of this approach is that the microbial community studies in which *S. mutans* was not mentioned or identified in the samples might be excluded in this review. We chose this approach out of practical reasons. In studies in which *S. mutans* was not mentioned or identified, accessing the raw sequencing data would be necessary to extract information on its presence. However, given the current data availability, this would be a very difficult task. For instance, in the current review, we asked for (additional) data of 18 articles. Only 11 responded to our requests, and 7 of these provided raw data. Seven included studies did not respond despite our repeated email requests. Moreover, while several studies made raw sequencing data available in public databases, they often lacked sufficient methodological details or sample information (metadata) required for (meaningful) comparisons. Therefore, we strongly recommend that future microbiome studies align caries diagnostic criteria and comprehensively register patient information, including diet habits, oral hygiene behavior, and detailed sequencing procedures. Improving data quality and ensuring open access will facilitate a more thorough assessment of the roles of *S. mutans* and other bacterial species in caries formation.

In conclusion, within the limit of the current systematic review, we analyzed the data from 19 clinical studies that not only evaluated the association between *S. mutans* level and caries experience but also examined composition of the microbial community in the same sample. The evidence indicated that a high prevalence and abundance of *S. mutans* are associated with increased caries experience. However, despite the strong association, the relative abundance of *S. mutans* in the microbial community was rather low. Therefore, while the cariogenic role of *S. mutans* in the oral ecosystem should be recognized, its actual function warrants further exploration.

## Author Contributions

D. Mazurel, contributed to conception, design, data acquisition, analysis, and interpretation, drafted and critically revised the manuscript; B.W. Brandt, contributed to data acquisition and interpretation, critically revised the manuscript; M. Boomsma, contributed to conception, design, data acquisition and interpretation, drafted the manuscript; W. Crielaard, M. Lagerweij, R.A.M. Exterkate, contributed to conception and design, critically revised the manuscript; D.M. Deng, contributed to conception, design, data interpretation, drafted and critically revised the manuscript. All authors gave final approval and agree to be accountable for all aspects of the work.

## Supplemental Material

sj-docx-1-jdr-10.1177_00220345241303880 – Supplemental material for Streptococcus mutans and Caries: A Systematic Review and Meta-Analysis Supplemental material, sj-docx-1-jdr-10.1177_00220345241303880 for Streptococcus mutans and Caries: A Systematic Review and Meta-Analysis by D. Mazurel, B.W. Brandt, M. Boomsma, W. Crielaard, M. Lagerweij, R.A.M. Exterkate and D.M. Deng in Journal of Dental Research
