# Supplementary material for: Streptococcus mutans and Caries: A Systematic Review and Meta-Analysis
Source: J Dent Res. 2025 Feb 2;104(6):594–603. doi: 10.1177/00220345241303880 (PMC12075887; doi:10.1177/00220345241303880)
Supplement: sj-docx-1-jdr-10.1177_00220345241303880 – Supplemental material for Streptococcus mutans and Caries: A Systematic Review and Meta-Analysis [file sj-docx-1-jdr-10.1177_00220345241303880.docx]

***Streptococcus mutans* and caries: A systematic review and meta-analysis**

Danuta Mazurel^1^, Bernd W. Brandt^1^, Marleen Boomsma^1^, Wim Crielaard^1^, Maxim Lagerweij^2^, Rob A. M. Exterkate^1^ and Dong Mei Deng^1^.

^1^ Department of Preventive Dentistry, Academic Center for Dentistry Amsterdam (ACTA), University of Amsterdam and VU University Amsterdam, Amsterdam, The Netherlands.

^2^ Department of Cariology, Academic Center for Dentistry Amsterdam (ACTA), University of Amsterdam and VU University Amsterdam, Amsterdam, The Netherlands.

**Supplementary files**

**Appendix Table 1.** Search strategy per database

| Database | Search strategy |
| --- | --- |
| Pubmed | ((demineralization) OR (caries) OR (carious) OR ("white spot") OR (dental decay)) AND ((DNA) OR (rDNA) OR (RNA) OR (rRNA) OR (16S) OR (sequencing) OR (PCR) OR (qPCR) OR (checkerboard) OR (microarray) OR (microbiome)) AND (mutans) |
| Cochrane | #1 mutans  #2 DNA  #3 rDNA  #4 RNA  #5 rRNA  #6 16S  #7 PCR  #8 qPCR  #9 sequencing  #10 checkerboard  #11 microarray  #12 #2 OR #3 OR #4 OR #5 OR #6 OR #7 OR #8 OR #9 OR #10 OR #11  #13 demineralization  #14 caries  #15 carious  #16 “dental decay”  #17 “white spot”  #18 #13 OR #14 OR #15 OR #16 OR #17  #19 #1 AND #12 AND 18 |
| Embase | #1 mutans  #2 DNA  #3 rDNA  #4 RNA  #5 rRNA  #6 16S  #7 PCR  #8 qPCR  #9 sequencing  #10 checkerboard  #11 microarray  #12 #2 OR #3 OR #4 OR #5 OR #6 OR #7 OR #8 OR #9 OR #10 OR #11  #13 demineralization  #14 caries  #15 carious  #16 “dental decay”  #17 “white spot”  #18 #13 OR #14 OR #15 OR #16 OR #17  #19 #1 AND #12 AND 18 |

**Appendix Table 2.** Reasons for exclusion after full-text screening and excluded articles (n=83)

| **Reason for exclusion** | **Articles** |
| --- | --- |
| **Not a clinical study (n=4)**  Case report, case series or review. | (Baik et al. 2013; Mei 2015; Pascal Andreu et al. 2021; Quivey et al. 2021) |
| **No comparison between caries-active and caries-free groups (n=23)** | (Darout et al. 2002; Dahlén, Nauclér, et al. 2010; Mantzourani et al. 2010; Soncini et al. 2010; Mannaa et al. 2013; Simón-Soro et al. 2014; ElSalhy et al. 2016; Aksit Blcak et al. 2017; Ali et al. 2017; Ribeiro et al. 2017; Abusleme et al. 2018; Yue et al. 2018; Inquimbert et al. 2019; Štšepetova et al. 2019; Gussy et al. 2020; Baumgartner et al. 2021; Huang et al. 2021; Wang et al. 2021; Yao and He 2021; Pang et al. 2022; Zhang et al. 2022; Yang et al. 2023) |
| **The microbiological data were not obtained by DNA/RNA based method or less than 40 bacterial species were reported (n=28)** | (Abe 1990; Acton et al. 1999; Tao et al. 2005; Barbosa da Silva et al. 2008; Nascimento et al. 2009; Dahlén, Konradsson, et al. 2010; Kanasi et al. 2010; Palmer et al. 2010; Jiang et al. 2011; Shimomura-Kuroki et al. 2011; Lu et al. 2015; Neves et al. 2015; Shimada et al. 2015; Al-Ahmad et al. 2016; Henne et al. 2016; Hu et al. 2016; Damé-Teixeira et al. 2018; Xiao et al. 2018; Bansal et al. 2020; Kalpana et al. 2020; O’Connell et al. 2020; Paqué et al. 2020; Zhang et al. 2020; Alia-García et al. 2021; Lee et al. 2021; Walther et al. 2021; Gómez-García et al. 2022) |
| **Systemic health and/or medication use (n=26)**  Studies including participants who had a systemic disease, were pregnant, or whose systemic health was not reported, and studies including participants who used medication (e.g., antibiotics) within the last month or whose medication status was not mentioned. | (Becker et al. 2002; Tanner et al. 2002; Corby et al. 2005; Aas et al. 2008; Preza et al. 2008; Preza et al. 2009; Gross et al. 2010; Kanasi et al. 2010; Gross et al. 2012; Simón-Soro et al. 2013; Wolff et al. 2013; Raner et al. 2014; Chen et al. 2015; Holgerson et al. 2015; Johansson et al. 2016; Agnello et al. 2017; Richards et al. 2017; Wolff et al. 2019; de Jesus et al. 2020; Ramadugu et al. 2020; Handsley-Davis et al. 2021; de Jesus et al. 2021; Pang et al. 2021; Bhaumik et al. 2022; Blostein et al. 2022; Lif Holgerson et al. 2023) |
| **Samples taken from foreign material (n=1)**  Study includes microbial samples taken from lesions directly adjacent to bonded orthodontic brackets. | (Tanner et al. 2012) |
| **Overlapping data (n=1)** | (Eriksson et al. 2018) |

**Appendix Table 3**. Quality assessment of included studies using the Joanna Briggs Institute (JBI) critical appraisal tool.

| **Study** | **Q1** | **Q2** | **Q3** | **Q4** | **Q5** | **Q6** | **Q7** | **Q8** | **Q9** | **Q10** | **JBI-score** ^a^ | **Risk of bias** |
| --- | --- | --- | --- | --- | --- | --- | --- | --- | --- | --- | --- | --- |
| Baker et al. 2021 | - | + | + | + | + | + | - | + | + | + | 8 | Medium |
| Belstrøm et al. 2017 | + | - | + | + | + | + | - | + | + | + | 8 | Medium |
| Dashper et al. 2019 | - | + | + | + | + | + | - | + | + | + | 8 | Medium |
| Eriksson et al. 2017 | + | + | + | + | + | + | + | + | + | + | 10 | Low |
| Garcia et al. 2021 | + | + | + | + | + | + | + | + | + | + | 10 | Low |
| Havsed et al. 2021 | + | + | + | + | + | + | n/a | + | + | + | 10 | Low |
| Hurley et al. 2019 | - | ? | + | + | + | - | - | + | + | + | 6 | **High** |
| Jiang et al. 2016 | + | + | + | + | + | + | n/a | + | + | + | 10 | Low |
| Kanasi et al. 2010 | + | + | + | + | + | + | n/a | + | + | + | 10 | Low |
| Kouidhi et al. 2014 | + | - | + | - | ? | + | - | + | + | + | 6 | **High** |
| Li et al. 2021 | + | + | + | + | + | + | n/a | + | + | + | 10 | Low |
| Ma et al. 2015 | ? | + | + | + | + | + | n/a | + | + | + | 9 | Medium |
| Qudeimat et al. 2021 | + | + | + | + | + | + | n/a | + | + | + | 10 | Low |
| Tang et al. 2022 | + | + | + | + | + | + | n/a | + | + | + | 10 | Low |
| Tanner et al. 2011 | + | + | + | + | + | + | n/a | + | + | + | 10 | Low |
| Wang et al. 2022 | + | + | + | + | + | + | n/a | + | + | + | 10 | Low |
| Wu et al. 2021 | + | + | + | + | + | + | n/a | + | + | + | 10 | Low |
| Wu et al. 2022 | + | + | + | + | + | + | - | + | + | ? | 8 | Medium |
| Yang et al. 2021 | + | + | + | + | + | + | n/a | + | + | + | 10 | Low |
| Zheng et al. 2017 | ? | + | + | + | + | + | ? | + | + | + | 8 | Medium |
| Zheng et al. 2018 | + | - | + | + | + | + | - | + | + | + | 8 | Medium |
| Zheng et al. 2021 | ? | ? | + | + | + | - | - | + | + | + | 6 | **High** |
|  | Q1 Were the groups comparable other than the presence of disease in cases or the absence of disease in controls?  Q2 Were cases and controls matched appropriately?  Q3 Were the same criteria used for identification of cases and controls?  Q4 Was exposure measured in a standard, valid and reliable way?  Q5 Was exposure measured in the same way for cases and controls?  Q6 Were confounding factors identified?  Q7 Were strategies to deal with confounding factors stated?  Q8 Were outcomes assessed in a standard, valid and reliable way for cases and controls?  Q9 Was the exposure period of interest long enough to be meaningful?  Q10 Was appropriate statistical analysis used? | | | | | | | | | | | |

^a^ Each of the 10 items (Q1 – Q10) evaluates a methodological feature as positive (“+”: Yes; “n/a”: not applicable), or negative (“-“: No, “?”: Unclear). A positive evaluation of an item counts for 1 point towards the total JBI-score of a study.

| **Study** | **Country** | **Subjects** | |  | **Sample** | |  | | **Detection method** | | | |  | | ***S. mutans* outcome ^c^** | | | | |
| --- | --- | --- | --- | --- | --- | --- | --- | --- | --- | --- | --- | --- | --- | --- | --- | --- | --- | --- | --- |
|  |  | **Number (CA/CF)**^a^ | **Age** |  | **Type**^b^ | **Collection method** |  | | **Community** | | **Target** |  | | | | **Prevalence** | | **Abundance** | |
| Baker, 2021 | USA | 49 (24/25) | 4-11 yr. |  | Saliva | Unstimulated, drooling/spitting | |  | | Sequencing | Whole genome | | |  | | | χ | | χ, σ |
| Belstrøm, 2017 | Denmark | 20 (10/10) | 22-50 yr. |  | Saliva | Stimulated, paraffin wax chewing | |  | | Sequencing | Whole genome | | |  | | | χ | | χ, σ, * |
| Dashper, 2019 | Australia | 131 (64/67) | 48 mo. |  | Saliva | Unstimulated, pipette/drooling | |  | | Sequencing | 16S rRNA region V4 | | |  | | | χ | | χ, σ |
| Eriksson, 2017 | Sweden | 154 (71/82) | 17 yr. |  | Saliva | Stimulated, paraffin wax chewing | |  | | Sequencing  (qPCR)^c^ | 16S rRNA region V3 – V4 | | |  | | | χ, * | | χ, * |
|  |  |  |  |  | Plaque | Pooled, all teeth | |  | |  |  |  |  |  | | |  |  |  |
| Garcia, 2021 | USA | 143 (70/73) | 40 mo. |  | Saliva | Unstimulated, pediatric mucus trap with vacuum pump | |  | | Sequencing | Whole genome | | |  | | | χ | | χ |
| Havsed, 2021 | Sweden | 40 (20/20) | 14-18 yr. |  | Plaque | Pooled, molars | |  | | Sequencing | 16S rRNA region V3 – V4 | | |  | | | χ | | χ |
| Jiang, 2016 | China | 40 (20/20) | 2-4 yr. |  | Saliva | Unstimulated, spitting | |  | | Sequencing | 16S rRNA region V3 – V4 | | |  | | | - | | χ, σ, * |
| Kanasi, 2010 | USA | 80 (39/41) | 2-6 yr. |  | Plaque | Pooled, molars (CF) or molars and molar carious lesions (CA) | |  | | Sequencing  (qPCR) | Full 16S rRNA | | |  | | | χ, * | | - |
| Li, 2021 | China | 130 (65/65) | 6-8 yr. |  | Saliva | Unstimulated, spitting | |  | | Sequencing | 16S rRNA region V1 – V3 | | |  | | | - | | * |
| Ma, 2015 | China | 40 (20/20) | 3-4 yr. |  | Saliva | Stimulated | |  | | HOMIM Microarray | 16S rRNA probes | | |  | | | χ, * | | * |
|  |  |  |  |  | Plaque | Pooled, all non-carious teeth | |  | |  |  |  |  |  | | |  |  |  |
| Qudeimat, 2021 | Kuwait | 128 (64/64) | 6-9 yr. |  | Plaque | Pooled, all teeth | |  | | Sequencing | 16S rRNA region V3 – V4 | | |  | | | χ | | χ, * |
| Tang, 2022 | China | 30 (15/15) | 42 mo. |  | Saliva | Unstimulated, drooling | |  | | Sequencing | Whole genome | | |  | | | - | | χ, * |
| Tanner, 2011 | USA | 85 (53/32) | 3-4 yr. |  | Plaque | Pooled, molars (CF) or molars and molar carious lesions (CA) | |  | | HOMIM Microarray  (qPCR) | 16S rRNA probes | | |  | | | χ, * | | - |
| Wang, 2022 | China | 20 (10/10) | 4-6 yr. |  | Saliva | Unstimulated, pipette | |  | | Sequencing | Full 16S rRNA | | |  | | | χ, σ, * | | χ, σ, * |
| Wu, 2021 | USA | 39 (21/18) | <6 yr. |  | Saliva | Unstimulated, saliva jet with suction pump | |  | | Sequencing | 16S rRNA region V1 – V3 | | |  | | | χ | | * |
|  |  |  |  |  | Plaque | Pooled, all teeth | |  | |  |  |  |  |  | | |  |  |  |
| Wu, 2022 | Thailand | 177 (98/79) | 3 yr. |  | Saliva | Stimulated, paraffin wax chewing | |  | | Sequencing | 16S rRNA region V3 – V4 | | |  | | | - | | * |
| Yang, 2021 | China | 30 (15/15) | 7-9 yr. |  | Saliva | Unstimulated, pipette | |  | | Sequencing | Full 16S rRNA | | |  | | | - | | * |
|  |  |  |  |  | Plaque | Pooled, deciduous and primary molars | |  | |  |  |  |  |  | | |  |  |  |
| Zheng, 2017 | China | 42 (21/21) | 19-31 yr. |  | Saliva | Stimulated | |  | | Sequencing  (qPCR) | 16S rRNA region V1 – V2 | | |  | | | χ | | χ, * |
| Zheng, 2018 | China | 31 (16/15) | 3-6 yr. |  | Plaque | Pooled, (CF) all teeth or (CA) all teeth and carious lesions | |  | | Sequencing | 16S rRNA region V3 – V4 | | |  | | | - | | χ, * |

**Appendix Table 4.** Study characteristics of 19 included studies.

^a^ The number of subjects listed in the table concerns the subjects who were caries-active (CA) or caries-free (CF).

^b^ Plaque refers to supragingival plaque

^c^ The bracket indicates that *S. mutans* in the samples were quantified by real-time PCR (qPCR), besides community sequencing.

^d^ χ: Mean values reported. σ: Standard deviation reported, *: significant difference between CA and CF groups was reported in the study.

**Appendix Figure 1.** The top-5 most-abundant genera in saliva or plaque samples reported in 15 of the 19 included studies. Relevant information could not be obtained from the remaining 4 studies (Kanasi et al. 2010; Tanner et al. 2011; Garcia et al. 2021; Li et al. 2021). The size of each section in the pie chart represents the number of studies that identified a specific genus as one of the top-5 genera. Only genera reported by at least 2 different studies for that same sample type are included in the pie chart. Genera reported in only one study are categorized as “other”.

**Appendix Table 5.** Diversity in community composition of the oral microbiome in saliva (n = 11) and plaque (n = 9) of CA versus CF groups.

|  | | **Number of studies** | | |
| --- | --- | --- | --- | --- |
|  |  | **Significant difference ^a^** | **No difference** | **Not reported** |
| **α-diversity** | **Saliva** | 1 (↑CF ^52^) | 8 | 5 |
|  | **Plaque** | 1 (↑CA ^37^) | 6 | 2 |
| **β-diversity** | **Saliva** | 5 | 3 | 6 |
|  | **Plaque** | 2 | 3 | 4 |

^a^ ↑ : higher (α) diversity reported in a study, with a reference. All other studies report a significant difference between CA and CF groups without a direction of the effect.
α-diversity: within sample diversity. β-diversity: between sample diversity. CA: caries-active. CF: caries-free.
